# Maladaptive Daydreaming: A Case report on phenomenology, differential diagnosis, and psychodynamic implications

**DOI:** 10.1192/j.eurpsy.2026.11724

**Published:** 2026-07-31

**Authors:** T. Jendričko, N. Sutara, A. Matić, P. Brečić

**Affiliations:** University Psychiatric Hospital Vrapče, Zagreb, Croatia

## Abstract

**Introduction:**

Maladaptive Daydreaming (MD) is a relatively new and still under-recognized phenomenon. It is characterized by prolonged and emotionally engaging immersion in highly structured fantasies. Unlike normal daydreaming, MD involves narrative scenarios with complex characters and plots that may extend into real life. Individuals often spend hours per day in such fantasies, typically triggered by music, films, or solitude. While MD may serve as a means of affect regulation or escape from reality, it often leads to emotional distress and impairment in daily functioning. Despite growing scientific interest, MD is not included in DSM-5 or ICD-11, which complicates recognition and treatment.

**Objectives:**

To present a clinical case of MD in a young adult and to discuss its phenomenology, differential diagnosis, and psychodynamic implications.

**Methods:**

We report a clinical case of maladaptive daydreaming and perform a non-systematic review of the literature.

**Results:**

A female university student in her early twenties was referred for outpatient psychotherapy due to emotional overwhelm. Since childhood, she had developed an ongoing imaginary narrative centered on a fictional partner. Over time, the story grew increasingly complex and included emotionally charged episodes. What stood out was the profound emotional involvement. The patient experienced these imagined events with striking intensity, as though they had truly occurred. Despite being academically successful and fulfilling her obligations, she spent several hours daily voluntarily immersing herself in these fantasies. Insight was preserved, and no psychotic symptoms were present. Differential diagnosis included obsessive–compulsive disorder, dissociative disorders, attention-deficit/hyperactivity disorder, and schizotypal personality disorder. Fantasies were voluntary, reality testing intact, and no intrusive obsessions were present, which all pointed to MD rather than psychosis or OCD. Epidemiological data suggest a prevalence of about 2.5% in the general population, more common in younger adults, and often associated with anxiety or depression. In this case, no major comorbidity was observed, yet the emotional intensity of fantasies pointed to clinical relevance.

**Conclusions:**

The case illustrates the core features of MD, such as a richly elaborated, voluntary fantasy life with significant emotional involvement that can affect real-life feelings. Psychodynamically, MD may function as a defense and affect regulation strategy that offers temporary relief and reinforces unmet relational needs. Recognition of MD is essential to avoid misdiagnosis and to guide psychotherapeutic interventions. Given the absence of official diagnostic criteria, detailed case reports remain crucial for clarifying MD’s nosological boundaries and supporting its potential inclusion in future diagnostic systems.

**Disclosure of Interest:**

None Declared

